# Data-Efficient and Explainable Multimodal Survival Prediction in NSCLC Using Deep Image Embeddings, Clinical Variables, and Gradient-Boosted Trees

**DOI:** 10.3390/diagnostics16121941

**Published:** 2026-06-22

**Authors:** Sevim Sahin, Adil Gursel Karacor

**Affiliations:** 1 Department of Electrical and Electronics Engineering, Faculty of Engineering and Natural Sciences, Fenerbahce University, Istanbul 34758, Türkiye; 2 Department of Industrial Engineering, Faculty of Engineering and Natural Sciences, Fenerbahce University, Istanbul 34758, Türkiye; adil.karacor@fbu.edu.tr

**Keywords:** NSCLC, survival prediction, multimodal learning, CT radiomics, deep image embeddings, gradient-boosted trees, explainable AI, small datasets

## Abstract

**Background/Objectives:** Survival prediction in non-small cell lung cancer (NSCLC) remains challenging, particularly in limited-sample settings where end-to-end deep learning models may suffer from limited generalization. This study aimed to develop a data-efficient, multimodal, and explainable framework integrating computed tomography (CT)-derived imaging information with clinical variables for NSCLC survival prediction. **Methods**: CT images, tumor segmentations, and clinical data from the publicly available NSCLC Radiomics (LUNG1) dataset (377 patients) were used. Tumor-focused regions were extracted using segmentation masks, and pretrained RadImageNet-InceptionV3 embeddings were obtained from the largest tumor-containing slice and neighboring-slice summaries. Deep imaging embeddings, engineered imaging features, and clinical variables were fused into a unified tabular representation. To improve robustness under limited-sample conditions, feature blocks were compressed using principal component analysis. CatBoost, XGBoost, and LightGBM models were trained on a development set and evaluated on a strictly held-out final validation set. **Results:** In three-class survival stratification, assigning censored/non-event patients to the upper survival group produced the strongest ordinal prognostic performance. Under the EX_PLUS_NON_EX_TOP setting, CatBoost achieved the best holdout score-based class C-index of 0.655. In continuous survival regression, LightGBM achieved the best holdout event-patient C-index of 0.576. Clinical variables provided the dominant prognostic signal, while compact deep image embeddings contributed complementary information, particularly in separating short- and long-survival groups. SHAP analysis confirmed contributions from both clinical and image-derived features. **Conclusions:** The proposed framework provides a proof-of-concept demonstration of a data-efficient and explainable image-to-tabular approach for NSCLC survival prediction under strict internal holdout validation. The results suggest that pretrained CT embeddings, clinical variables, gradient-boosted trees, and SHAP-based interpretation can be combined in a feasible, limited-sample survival modeling pipeline, while external validation remains necessary before clinical translation.

## 1. Introduction

Lung cancer is still the leading cause of cancer-related mortality worldwide, and non-small cell lung cancer (NSCLC) accounts for nearly 85% of all lung cancer cases [1]. Although imaging, radiotherapy, and systemic treatments have improved over time, survival outcomes in NSCLC remain highly variable. Patients with the same clinical stage may still experience very different disease courses, which underlines the need for more individualized survival prediction models [2].

The tumor-node-metastasis (TNM) staging remains the standard basis for clinical risk stratification, but it cannot fully reflect the biological and imaging-related heterogeneity of NSCLC tumors [3]. Patient-level variables such as age, tumor stage, and histological subtype are also important for prognosis. However, these factors alone do not always explain the large variation in survival observed across patients [2,4]. For this reason, imaging-based prognostic models have become an important area of research. Radiomics studies have shown that handcrafted features extracted from computed tomography (CT) images can describe tumor heterogeneity and provide prognostic information in NSCLC [5,6]. At the same time, radiomics workflows can be affected by differences in image acquisition, segmentation quality, and feature selection, which may reduce reproducibility across datasets [7].

Deep learning methods, particularly convolutional neural networks (CNNs), have expanded imaging-based survival modeling by learning image features directly from CT data [8,9]. While these models can learn complex spatial representations, they typically require large, annotated datasets and may overfit in small-cohort settings. In addition, their limited interpretability remains a challenge for clinical use [10].

Recent studies have therefore shifted toward hybrid and multimodal modeling strategies, in which imaging-derived features are integrated with clinical variables to improve prognostic performance beyond single-modality approaches [9,11]. Related data-efficient strategies have also been reported in other medical imaging domains, where learned image representations are combined with structured descriptors [12]. However, many available frameworks remain dependent on complex model architectures, extensive feature engineering, or larger datasets, making their translation to small-sample settings less straightforward.

In this study, we developed a data-efficient and interpretable multimodal strategy for NSCLC survival prediction. Tumor-focused CT information was represented through compact pretrained radiological embeddings and integrated with structured clinical variables in a tabular learning pipeline. Performance was evaluated on a separate validation set reserved from the same dataset to obtain a conservative estimate of generalization. The analysis included both continuous survival regression and survival stratification, with SHAP-based interpretation used to examine global feature contributions and patient-level model behavior.

## 2. Related Work

Quantitative imaging-based survival prediction in NSCLC has been widely studied, with radiomics approaches forming the basis of early work in this field. Handcrafted features extracted from CT images have been used together with statistical and machine learning models, including Cox regression and random survival forests, to predict survival outcomes [5,13,14]. While these methods have shown promising results, they are often sensitive to imaging conditions, segmentation accuracy, and feature selection procedures, which can affect reproducibility [7,15].

To overcome these limitations, deep learning-based approaches have been increasingly adopted. CNN-based models have been used to learn hierarchical feature representations directly from imaging data, including both 3D volumetric approaches and more computationally efficient 2D or 2.5D slice-based strategies [8,9]. Slice-based methods, such as using the largest tumor slice together with adjacent slices, offer a practical alternative to full 3D modeling while still capturing relevant tumor information [16]. However, these models generally require large annotated datasets and are often difficult to interpret [10].

Hybrid and multimodal approaches have gained attention as a way to combine imaging and clinical information. Several studies have shown that integrating clinical variables such as age, tumor stage, and histology with imaging-derived features can improve predictive performance in NSCLC survival prediction [11,17,18]. Nevertheless, many existing multimodal models rely on complex architectures or extensive feature engineering pipelines, which may limit their applicability in real-world clinical settings, particularly when data are limited [10,18].

More recently, there has been growing interest in data-efficient and interpretable modeling strategies. Tree-based ensemble methods, such as gradient-boosted decision trees, have demonstrated strong performance in structured tabular settings and can effectively model non-linear relationships between heterogeneous feature types [19,20]. When combined with compact imaging representations, these approaches provide a practical balance between predictive performance, computational efficiency and interpretability.

Despite these advances, there remains a need for simplified and robust multimodal frameworks that can effectively integrate imaging and clinical data while maintaining computational efficiency and applicability to limited-sample datasets. The present study addresses this gap by proposing a compact image-to-tabular multimodal framework that combines pretrained CT embeddings with clinical variables and evaluates performance on a separate validation set.

## 3. Materials and Methods

### 3.1. Dataset and Outcome Definition

This study used the publicly available NSCLC-Radiomics (LUNG1) dataset, originally released through The Cancer Imaging Archive (TCIA) and linked to the radiomics study of Aerts et al. [21]. In this paper, a total of 377 patients were included. The available data comprised three main modalities: pretreatment thoracic CT images, tumor segmentation masks, and clinical/tabular variables. The CT images and segmentation masks were used for tumor-focused image analysis and feature extraction, whereas the clinical variables were used to represent patient-level prognostic information in tabular form.

The primary outcome of interest was overall survival time in days. In line with the main objective of the study, survival prediction was formulated primarily as a continuous regression problem rather than being restricted to survival-group classification. Patients with an observed event were treated as the main cohort for continuous survival modeling and event-based concordance analysis. Patients without an observed event were excluded from the primary continuous survival regression analysis; in a separate three-class survival stratification setting, these non-event cases were assigned to the upper survival group, leading to improved ordinal prediction performance. This design allowed the study to preserve a real-valued survival prediction framework while also enabling comparison with the survival-group formulations that are commonly reported in the literature.

### 3.2. Data Partitioning and Strict Final Validation Protocol

To obtain a more reliable estimate of generalization performance, the dataset was partitioned into a development set and a strictly held-out final validation set. From the full cohort of 377 patients, 302 patients were assigned to the development set, and 75 patients were reserved for final validation. Within the event subgroup used for primary continuous survival regression, this corresponded to 268 event cases in the development set and 67 event cases in the final validation set. The remaining non-event cases were retained separately and were incorporated only in the supporting three-class survival stratification setting.

All model selection, hyperparameter tuning, and intermediate performance comparisons were performed exclusively within the development set. In this stage, 5-fold cross-validation was applied only to the development data. The final validation set was not used at any point during model development, cross-validation, hyperparameter search, feature selection decisions, or model comparison. The final validation set was used only after the modeling pipeline had been finalized, for final performance evaluation and post hoc interpretation.

To avoid information leakage, all data-dependent transformations were fitted using the relevant training portion of the development data and then applied to the corresponding internal validation fold or to the final validation set without refitting. This rule was followed throughout the pipeline, including feature-space transformations and dimensionality reduction steps. As a result, the reported final validation results reflect a stricter and more realistic evaluation setting than the train/test-only protocols commonly used in related small-dataset studies.

**Figure 1:**
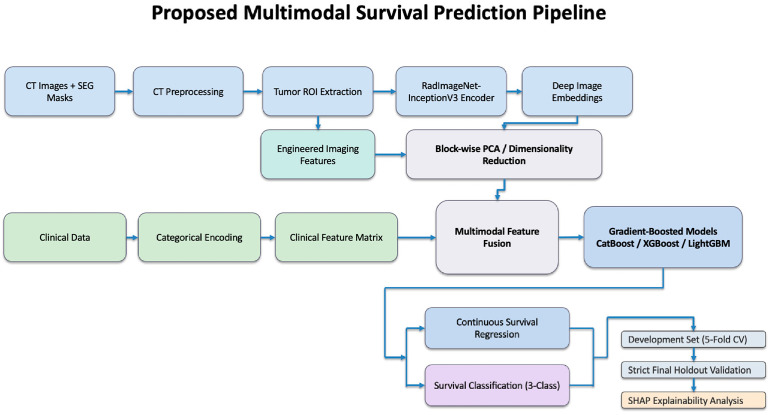
Proposed multimodal survival prediction pipeline.

An overview of the complete multimodal survival prediction workflow, including data processing, feature extraction, fusion, modeling, and evaluation stages, is presented in Figure 1.

### 3.3. CT Image Preprocessing and Tumor ROI Extraction

The CT image data were processed from the original DICOM files. For each patient, the corresponding CT series was identified, and the axial slices were ordered using available DICOM metadata, primarily the instance number and slice position information. Pixel intensities were converted into Hounsfield Units (HU) using the rescale slope and intercept values stored in the DICOM headers. For visualization and tumor localization, CT images were displayed using a lung-window setting to improve the visibility of lung parenchyma and tumor regions.

**Figure 2:**
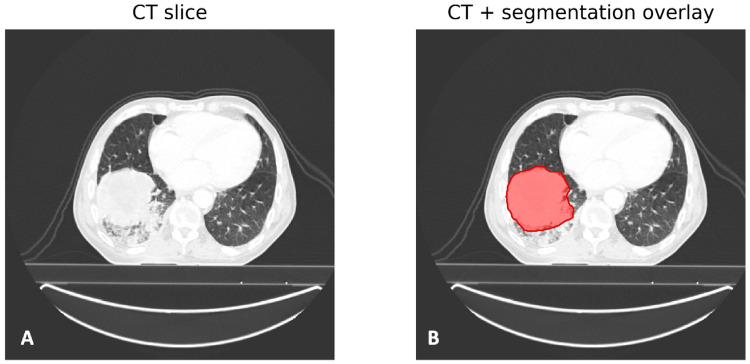
CT slice (**A**) with corresponding tumor segmentation overlay (**B**).

Tumor segmentation masks were obtained from the available DICOM segmentation objects and mapped to the corresponding CT slices. The alignment between CT images and segmentation frames was performed using the referenced CT slice identifiers stored in the segmentation metadata. This ensured that each segmentation mask was placed on its correct axial CT slice. An example CT slice and the corresponding tumor segmentation overlay are shown in Figure 2.

**Figure 3:**
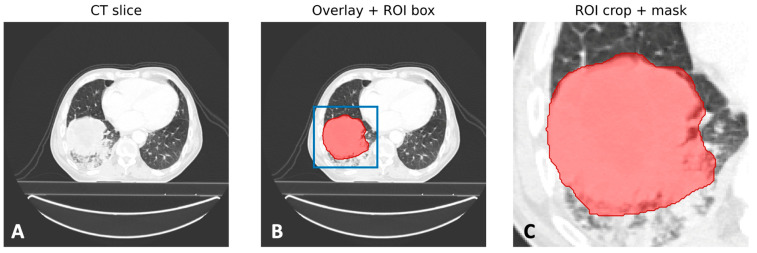
Tumor ROI extraction process from CT images. Original axial CT slice (**A**), tumor segmentation overlay and ROI bounding box (**B**), and cropped tumor ROI with applied segmentation mask used for analysis (**C**).

For each patient, the tumor-containing slice with the largest segmented tumor area was identified as the primary representative slice for image-based feature extraction. In addition, neighboring or top-ranked tumor-containing slices were retained where applicable to support multi-slice summary embeddings in the subsequent feature extraction stage. A bounding box was computed around the tumor mask on the selected slices and expanded with an additional margin to preserve local anatomical context around the lesion. The resulting tumor-centered regions of interest (ROIs) were cropped from the CT slices and used in the subsequent deep embedding extraction step. Figure 3 illustrates the ROI extraction process, including the original CT slice, the segmentation overlay with the ROI box, and the final cropped tumor region.

### 3.4. Deep Image Embedding Extraction Using RadImageNet-InceptionV3

After tumor ROI extraction, deep image representations were obtained using a pretrained RadImageNet-InceptionV3 convolutional neural network [22]. Unlike models pretrained on natural images, RadImageNet provides domain-specific representations learned from large-scale radiological imaging data, making it more suitable for medical imaging applications.

In this study, the InceptionV3 backbone was used as a fixed feature extractor. The classification head was removed, and the global average pooled output of the final convolutional layers was used as a compact embedding vector for each input ROI. The pretrained weights were loaded from a RadImageNet InceptionV3 model, and no further fine-tuning was performed to reduce overfitting risk under the small-data setting. The embedding extraction pipeline was implemented using TensorFlow (version 2.21.0) [23].

Before being passed to the network, tumor-centered ROI images were converted from Hounsfield Units to normalized intensity values using a lung windowing scheme. The resulting grayscale images were replicated across three channels and resized to the required input resolution of the InceptionV3 architecture (299 × 299). Standard preprocessing associated with the InceptionV3 model was then applied.

Two complementary embedding strategies were generated for each patient. First, a single-slice representation was obtained from the tumor-containing slice with the largest segmented tumor area. Second, a multi-slice representation was constructed by extracting embeddings from the neighboring slices (best slice ±1) and computing their mean. This approach allows the model to capture limited volumetric context while maintaining a compact representation and avoiding the complexity of full 3D modeling.

The resulting embedding vectors were not used directly as high-dimensional predictors. Instead, they were integrated into a multimodal tabular representation together with clinical variables and engineered imaging features. This design allows the framework to leverage deep radiological information while preserving robustness, interpretability, and suitability for small-sample learning.

### 3.5. Engineered Imaging Features

In addition to deep image embeddings, a set of low-dimensional engineered imaging features was extracted from the tumor segmentation masks and the corresponding CT regions. These features were designed to provide simple, interpretable descriptors of tumor size, shape, and local image characteristics. Unlike deep embeddings, which capture high-level visual representations, the engineered features summarize explicit properties of the segmented tumor and its surrounding region.

For each patient, geometric features were derived from the tumor mask and the extracted ROI. These included descriptors related to the segmented tumor area, ROI size, bounding-box dimensions, aspect ratio, and the relative proportion of the tumor within the cropped ROI. Such features provide direct information about tumor extent and spatial compactness on the selected CT slice. Where multi-slice information was used, summary descriptors were also derived from the tumor-containing slices to represent variation in tumor area across neighboring slices.

Basic statistical image descriptors were also computed from the CT intensity values within the tumor-centered region. These features were intended to capture simple local intensity patterns after HU conversion and lung-window preprocessing. Together, the geometric and statistical descriptors provided an interpretable image-derived feature block that complemented the more abstract RadImageNet-InceptionV3 embeddings.

The engineered imaging features were not intended to replace deep image embeddings. Instead, they were included as an additional compact feature group in the multimodal tabular dataset. This allowed the model to combine explicit tumor-level descriptors with learned deep representations and clinical variables in the final prediction pipeline.

### 3.6. Clinical Tabular Data Preprocessing

Clinical variables were processed as the main tabular component of the multimodal prediction framework. The available clinical descriptors included demographic and disease-related variables such as age, sex, tumor stage information, overall stage, and histology. These variables were used to represent patient-level prognostic information that could complement the CT-derived image features.

Before model training, clinical variables were checked for consistency and converted into a machine-learning-ready tabular format. Numerical variables were kept as continuous predictors, whereas categorical variables were encoded using integer-based label encoding. This encoding strategy was selected because the downstream models were gradient-boosted decision trees, which can handle non-linear splits over encoded tabular variables without requiring one-hot expansion in this small-sample setting.

A very small number of missing clinical values were present in the dataset. Missingness was minimal, with fewer than 1% missing entries in the affected clinical variables. These sparse missing entries were imputed using the most frequent value of the corresponding variable. This simple imputation strategy was preferred because the missingness level was very low and because more complex imputation methods could introduce unnecessary instability in a limited-data setting. In line with the validation protocol, imputation values were estimated from the training portion of the data and then applied to the corresponding validation data.

The final clinical feature block was kept intentionally compact. Rather than generating a large number of derived clinical variables, the preprocessing focused on preserving clinically meaningful predictors and avoiding unnecessary feature expansion. The processed clinical variables were then combined with deep image embeddings and engineered imaging features in the multimodal fusion stage.

### 3.7. Multimodal Feature Fusion and Block-Wise Dimensionality Reduction

The processed clinical variables, engineered imaging features, and RadImageNet-InceptionV3 embeddings were combined into a single multimodal tabular representation. This fusion strategy allowed the final prediction models to jointly leverage patient-level clinical information and CT-derived imaging information within a unified structured learning framework.

Each deep embedding vector consisted of 2048 features, corresponding to the global average pooled output of the InceptionV3 backbone. Given the limited sample size of the dataset, this high-dimensional representation required dimensionality reduction before being used in downstream modeling.

Instead of applying a single dimensionality reduction step to the entire feature set, a block-wise principal component analysis (PCA) strategy was used. This approach enabled each feature group to be compressed independently while preserving the structure of the multimodal pipeline.

PCA was applied to the image-derived feature blocks, including the largest-slice deep embeddings, the multi-slice mean embeddings, and the engineered imaging features. The clinical variables were kept in their original processed form because they were already low-dimensional and clinically interpretable. This design prevented the high-dimensional embedding blocks from dominating the feature space while preserving the direct interpretability of the clinical predictors.

The final fused feature set therefore consisted of the processed clinical variables together with the reduced image-derived components. In the final M3 configuration, the tabular representation included seven clinical variables and five PCA-derived imaging components: one component from the engineered imaging feature block, two components from the largest-slice RadImageNet embedding block, and two components from the best-slice ±1 mean embedding block. This configuration was fixed a priori as a compact, small-data-oriented design choice to preserve information from each image-derived feature source while avoiding excessive dimensionality relative to the cohort size. The resulting representation formed a compact 12-feature multimodal dataset for downstream modeling. This compact representation was critical for reducing overfitting risk and improving robustness under the limited-sample setting of the study.

The number of PCA components was fixed a priori and was not selected using the final validation set. All dimensionality reduction steps were fitted strictly within the development data in accordance with the validation protocol. During cross-validation, PCA was fitted only on the training folds and then applied to the corresponding internal validation folds. For the final evaluation, the PCA transformations were fitted on the full development set and then applied to the separate final validation cohort without refitting. This ensured that the fusion and dimensionality reduction steps did not introduce information leakage.

### 3.8. Gradient-Boosted Tree Regression Models

The primary survival prediction task was formulated as a continuous regression problem. Three gradient-boosted tree algorithms were evaluated: CatBoost [24], XGBoost [25], and LightGBM [26]. These models were selected because gradient-boosted trees are strong predictors for structured tabular data and can handle non-linear interactions between clinical variables, engineered imaging descriptors, and reduced deep embedding components.

Because survival times were highly skewed, the regression target was transformed using a logarithmic transformation before model training. Specifically, models were trained to predict log-transformed survival time, using log(1 + survival_days). Predicted values were then transformed back to the original day scale when computing error-based metrics such as root mean squared error (RMSE) and mean absolute error (MAE). Concordance-based metrics were computed from the predicted survival ordering among event patients. Although the models were trained on log(1 + survival_days), the C-index is rank-based and invariant to monotonic transformations; therefore, the same ordering is obtained from log-scale predictions and back-transformed survival-day predictions.

Hyperparameter tuning was performed only within the development set using 5-fold cross-validation with a fixed random seed of 1919. For each algorithm, a compact search over clinically reasonable and small-data-appropriate hyperparameter ranges was conducted. The tuned parameters included the number of trees or boosting iterations, learning rate, tree depth, regularization strength, and subsampling rate, where applicable. No early stopping was used during training. Detailed hyperparameter settings and the main software environment are provided in Supplementary Table S1. Model configurations were compared using cross-validated survival ranking performance on the development set. The final holdout validation set was not used at any stage of model training, hyperparameter tuning, model selection, or stopping decisions.

Model selection was based primarily on cross-validated survival ranking performance, with error-based regression metrics used as secondary indicators of stability. This was important because the main objective was not only to minimize absolute prediction error, but also to preserve clinically meaningful ordering of patients by survival outcome. After hyperparameter selection, each model was retrained on the full development set and evaluated once on the separate final validation set.

This modeling strategy allowed the study to combine deep image-derived representations and clinical variables within a robust tabular learning framework, without relying on end-to-end deep survival modeling. This was particularly important given the limited sample size of the dataset.

### 3.9. Auxiliary Survival Stratification Analysis

Although the main objective of the study was continuous survival regression, an auxiliary survival stratification analysis was also performed to support comparison with classification-based survival prediction approaches commonly used in the literature. In this setting, survival time was converted into a three-class ordinal outcome representing short-, intermediate-, and long-survival groups.

The three survival classes were defined using fixed survival-day thresholds derived from the empirical distribution of observed survival times in the development event cohort. Specifically, the thresholds of 303 and 703 days corresponded to tertile-based cut-points derived from event patients in the development set. These cut-points were used to define short-, intermediate-, and long-survival categories: patients with survival time below 303 days were assigned to the short-survival group, patients between 303 and 703 days were assigned to the intermediate-survival group, and patients above 703 days were assigned to the long-survival group. The thresholds were determined only from the development data and then kept fixed when applied to the final holdout validation set, avoiding information leakage from the final validation cohort. Therefore, these cut-points should be interpreted as empirical stratification thresholds for the auxiliary analysis rather than as universally established clinical NSCLC survival cut-offs.

Two analysis scopes were considered. In the EX_ONLY setting, only patients with an observed event were included. This setting was aligned with the primary continuous regression framework, where survival time was directly observed. In the EX_PLUS_NON_EX_TOP setting, non-event/censored patients were additionally included by assigning them to the upper survival group. This was used only as a supporting ordinal stratification analysis, not as the primary regression target. The purpose was to evaluate whether censored patients, who did not experience the event during the observed follow-up period, could improve long-survival group separation in a classification-style prognostic setting.

Two complementary stratification approaches were evaluated. First, the continuous regression outputs were converted into survival groups using the same class thresholds, allowing the regression models to be assessed also from an ordinal survival-group perspective. Second, direct three-class classification models were trained using CatBoost, XGBoost, and LightGBM to predict the survival group labels directly. This allowed comparison between the primary regression-based framework and the more common survival-group classification formulation.

This auxiliary analysis was therefore used to assess whether the proposed multimodal feature representation could support clinically meaningful survival grouping in addition to real-valued survival prediction. However, the continuous survival regression task remained the primary modeling framework of the study.

### 3.10. Evaluation Metrics

Model performance was evaluated from both continuous regression and auxiliary survival stratification perspectives. Since the primary task was real-valued survival prediction, the main evaluation focused on regression-based metrics computed on patients with observed events. The principal ranking metric was Harrell’s concordance index (C-index), calculated between observed survival days and predicted survival values among event patients. Because the model output represented predicted survival time rather than risk, higher predicted values were interpreted as longer expected survival.

For continuous regression, error-based metrics were also reported to quantify the absolute deviation between predicted and observed survival times. These included RMSE, MAE, root mean squared logarithmic error (RMSE-log), mean absolute logarithmic error (MAE-log), and Pearson correlation. RMSE and MAE were computed after transforming predictions back to the original survival-day scale, whereas the logarithmic metrics were computed on the log-transformed survival scale. These metrics were used as complementary indicators, while the C-index remained the main metric for assessing survival ranking performance.

For the auxiliary three-class survival stratification analysis, classification metrics were reported, including accuracy, macro-averaged F1-score, and log-loss. Accuracy measured the overall proportion of correctly classified survival groups, whereas macro-F1 was included to account for performance across all three classes without letting the largest class dominate the evaluation. Log-loss was used to assess the quality of probabilistic class predictions.

In addition to standard classification metrics, several ordinal C-index variants were computed to evaluate whether the predicted survival groups preserved the correct survival ordering. First, a class-based C-index was calculated using the ordered true survival classes and either the predicted class labels or probability-derived class scores. Second, the event-patient survival-days C-index was calculated by comparing observed survival days with the predicted ordinal class outputs among event patients. These metrics were used to assess whether the classification-style models produced clinically meaningful short-, intermediate-, and long-survival ordering.

All metrics were computed separately for development cross-validation and for the separate final validation set. The final validation metrics were used as the main evidence of generalization performance, because this set was not used during model training, hyperparameter tuning, feature processing decisions, or model selection.

### 3.11. SHAP-Based Explainability Analysis

SHAP-based (SHapley Additive exPlanations) explainability analysis was performed to interpret the predictions of the final CatBoost survival stratification model. SHAP [27] values were used to quantify the contribution of each feature to the model output and to assess how clinical variables and image-derived components influenced survival-group predictions. The analysis focused on the three-class survival stratification setting, where the model predicted short-, intermediate-, and long-survival groups.

To avoid information leakage, the explainability pipeline followed the same development and final validation protocol used for model training. The CatBoost model was trained only on the development set using the final M3 feature representation. The preprocessing steps, including imputation, scaling, and block-wise PCA, were fitted on the development data and then applied to the final holdout validation set without refitting. SHAP values were then computed for the final holdout predictions using CatBoost’s native SHAP implementation.

Feature-level SHAP importance was computed globally by averaging the absolute SHAP values across final holdout patients and survival classes. In addition, class-wise SHAP summaries were generated to evaluate whether different predictors contributed differently to short-, intermediate-, and long-survival predictions. This was important because a feature may contribute strongly to separating long-survival patients even if its average effect across all classes is more moderate.

To assess the relative contribution of different information sources, features were also grouped into broader blocks: clinical variables, engineered imaging components, largest-slice embedding components, and multi-slice mean embedding components. Block-level SHAP importance was calculated by aggregating the feature-level SHAP contributions within each group. This allowed the analysis to compare the explanatory contribution of clinical information and CT-derived image representations at a higher level.

Local explanations were also generated for individual final holdout patients. For each patient, the most influential features for the predicted survival class were identified, including whether each feature pushed the prediction toward or away from that class. These local explanations were used to inspect representative correct and incorrect predictions and to better understand how the model combined clinical and image-derived information in individual cases.

Overall, the SHAP analysis was used not only to rank individual predictors, but also to evaluate whether the multimodal design provided interpretable contributions from both clinical and imaging feature blocks. This supported the explainability objective of the proposed framework while keeping the final prediction model within a structured tabular learning setting.

### 3.12. Computational Complexity and Runtime Analysis

The proposed pipeline consists of two main stages: deep feature extraction using a pretrained RadImageNet-InceptionV3 network and downstream modeling using gradient-boosted tree algorithms.

The embedding extraction step is performed once per tumor-focused ROI and scales approximately linearly with the number of processed patients and slices. Since the convolutional network is used only in inference mode without fine-tuning, this stage avoids the computational cost of end-to-end CNN training. The use of a representative tumor-containing slice and a small neighboring-slice summary also reduces the computational burden compared with full 3D or end-to-end volumetric deep learning approaches.

After feature extraction, the remaining steps operate on a compact tabular representation. Block-wise PCA is applied to relatively small feature matrices, and the final CatBoost, XGBoost, and LightGBM models are trained on low-dimensional tabular inputs. This design is intended to be suitable for limited-sample settings, where compact tabular modeling can be more practical than training large deep neural networks from scratch. In our experiments, the complete pipeline could be executed successfully on standard CPU hardware without requiring end-to-end CNN training.

However, no dedicated runtime, memory-consumption, or hardware benchmarking experiment was performed in the present study. Therefore, the computational efficiency of the proposed framework should be interpreted as a practical design consideration rather than as a formally benchmarked result. Future work should include systematic runtime and memory profiling across different hardware settings, including CPU-only and GPU-assisted implementations.

## 4. Results

### 4.1. Cohort and Split Summary

A total of 377 patients from the NSCLC-Radiomics (LUNG1) cohort were included in the analysis. Of these, 335 patients had an observed event, while 42 patients were non-event/censored cases. The dataset was divided into a development set used for model development and cross-validation, and a strictly held-out final validation set used only for final performance assessment. Baseline clinical characteristics of the two subsets are summarized in Table 1.

The development and final validation sets showed similar event/non-event proportions and broadly comparable age and stage distributions. As expected in a limited-size cohort, some distributional differences were present between the two subsets. However, this split preserved the main event-patient structure of the dataset while keeping a separate final validation cohort for unbiased evaluation of generalization performance.

### 4.2. Primary Continuous Survival Regression

The primary prediction task was evaluated as continuous survival regression on patients with observed events. Table 2 summarizes the development cross-validation and final holdout validation results for the three gradient-boosted tree models. The main evaluation metric was the C-index, while RMSE, MAE, logarithmic error metrics, and Pearson correlation were reported as complementary regression indicators.

Across the development cross-validation setting, all three models showed comparable survival ranking performance, with C-index values ranging from 0.596 to 0.605. LightGBM achieved the highest development C-index (0.605) and also produced the lowest development RMSE and MAE. On the separate final validation set, LightGBM again obtained the best C-index (0.576), followed by CatBoost (0.564) and XGBoost (0.556). Although error-based metrics increased on the final holdout set, the models preserved a consistent survival-ranking signal under a stricter evaluation protocol.

These results show that the proposed multimodal tabular framework can perform real-valued survival prediction under limited-data conditions. Unlike survival-group classification alone, this analysis evaluates the models directly on continuous survival time in days, while the auxiliary stratification results are presented separately in the following section.

It is important to note that many previously reported results in the literature are based on development-stage evaluation protocols, such as cross-validation or train/test splits, where model selection and hyperparameter tuning may be performed on the same data used for performance reporting. In contrast, the results reported in this study are obtained from a strictly held-out final validation set, which was not involved in any stage of model development. As a result, the reported performance provides a more conservative but reliable estimate of generalization.

An observed-versus-predicted diagnostic plot for the LightGBM continuous regression model on final holdout event patients is provided in Supplementary Figure S1B. Because survival time was highly skewed, the plot is shown on a log(1 + days) scale.

### 4.3. Auxiliary Three-Class Survival Stratification

To facilitate comparison with survival-group-based approaches commonly reported in the literature, an auxiliary three-class survival stratification analysis was performed. The models were evaluated under two settings: EX_ONLY, where only patients with observed events were included, and EX_PLUS_NON_EX_TOP, where non-event/censored patients were assigned to the upper survival class.

The resulting class distributions are shown in Table 3. In the EX_ONLY setting, the three survival classes were well balanced because the thresholds were derived from tertiles of observed survival time in the development event cohort. In the EX_PLUS_NON_EX_TOP setting, the long-survival class became larger by design because non-event/censored patients were assigned to the upper survival group.

The corresponding model performance results are summarized in Table 4.

In the EX_PLUS_NON_EX_TOP setting, the development-stage results showed meaningful ordinal separation between survival groups. Using probability-derived ordinal class scores, CatBoost and XGBoost achieved class-based C-index values of 0.690 and 0.688, respectively, while LightGBM achieved 0.673. These results are particularly relevant for comparison with previous studies that report performance from cross-validation or train/test-style development protocols. However, they should be interpreted as development-stage performance rather than as final unbiased evidence of generalization.

On the separate final validation set, the score-based class C-index remained meaningful, with values of 0.655 for CatBoost, 0.645 for XGBoost, and 0.639 for LightGBM in the EX_PLUS_NON_EX_TOP setting. LightGBM achieved the highest final holdout accuracy (0.467) and macro-F1 score (0.429), while CatBoost achieved the highest score-based class C-index. These results suggest that the proposed multimodal representation can support survival-group prediction with ordinal consistency under a strict internal holdout validation protocol.

The inclusion of non-event/censored patients in the upper survival class improved the auxiliary stratification results compared with the EX_ONLY setting. This is consistent with the interpretation that patients without an observed event during follow-up provide useful information for defining the long-survival group. In contrast, excluding these cases reduces the effective sample size and weakens the representation of longer-survival outcomes.

When evaluated against observed survival days among event patients, the days-based C-index values remained in a narrower range across models. This indicates that the auxiliary classification models also preserved some continuous survival ordering among event patients. Nevertheless, this analysis was not intended to replace the primary regression framework. Instead, it provides a complementary survival-group perspective and enables a fairer comparison with classification-style survival prediction studies in the literature.

A calibration-style diagnostic plot for the CatBoost EX_PLUS_NON_EX_TOP stratification model on the final holdout set is provided in Supplementary Figure S1A. The plot compares predicted class probabilities with observed class frequencies in probability bins.

### 4.4. Regression-Derived Stratification vs. Direct Multiclass Classification

To clarify the difference between the primary regression framework and classification-style survival prediction, regression-derived stratification was compared with direct three-class classification. In the regression-derived setting, the models first predicted continuous survival time in days, and these predictions were then converted into the same three survival groups using the predefined thresholds. In the direct classification setting, the models were trained to predict the three survival classes directly. The final holdout validation results are summarized in Table 5.

As expected, the direct multiclass classifiers generally achieved higher accuracy and macro-F1 scores, because they were optimized directly for class-label prediction. In the EX_PLUS_NON_EX_TOP setting, LightGBM improved from an accuracy of 0.307 and macro-F1 of 0.225 in the regression-derived setting to 0.467 and 0.429 in the direct classification setting. Similar improvements were observed for CatBoost and XGBoost.

However, the regression-derived approach remained important because it represents the ordinal counterpart of the primary continuous survival regression framework. When continuous predicted survival values were used as ordinal scores, regression-derived stratification achieved competitive class-based C-index values. For example, in the EX_PLUS_NON_EX_TOP setting, LightGBM achieved a class C-index of 0.654, which was comparable to the score-based C-index values obtained by direct classification models (0.639–0.655).

These findings show that direct multiclass classification is better suited when the objective is survival-group assignment, while regression-derived stratification preserves the link to real-valued survival prediction. Therefore, the two analyses should not be interpreted as substitutes, but as complementary views of the same prognostic modeling problem.

### 4.5. Comparison with Previous Studies

To place the proposed framework in context, Table 6 summarizes selected recent studies on NSCLC survival prediction and multimodal cancer prognosis together with the present results. This table is intended as a contextual overview rather than a direct head-to-head performance benchmark, because the studies differ in cohort size, imaging modality, task formulation, model architecture, and validation design. Therefore, the reported C-index values should be interpreted together with the evaluation protocol shown in the table.

For a literature-comparable comparison, the most relevant result from the present study is the EX_PLUS_NON_EX_TOP three-class survival stratification setting, where non-event patients were included in the upper survival class. This setting is closer to the risk-stratification and survival-group formulations commonly used in previous prognostic modeling studies [11,18,28]. Under this development-stage setting, our model achieved a score-based class C-index of 0.690 with CatBoost, 0.688 with XGBoost, and 0.673 with LightGBM. These values are within the range of several recent multimodal survival prediction studies, despite using only 377 patients and a compact tabularized feature representation.

In addition to this literature-comparable development-stage result, the present study also reports performance on a strictly held-out final validation set. In this stricter setting, the best EX_PLUS_NON_EX_TOP score-based class C-index was 0.655. This final validation cohort was not used during feature processing, hyperparameter tuning, model selection, or cross-validation. Therefore, this result should be interpreted as a more conservative estimate of generalization performance.

The primary continuous regression result is reported separately because it addresses a different and more demanding task: direct prediction of survival time in days among event patients. In that setting, the best final holdout C-index was 0.576. This value should not be compared directly with classification-style or risk-stratification C-index values, but it supports the study’s main methodological claim that survival prediction was not reduced only to coarse survival-group classification.

For completeness, broader multimodal prognostic modeling studies that integrate multiple data sources beyond CT imaging, such as pathology and genomics, have also reported competitive C-index values in the range of approximately 0.67–0.70 [29], although these studies are not directly comparable due to differences in data modalities and problem formulation.

Development-stage results are comparable to commonly reported train/test-style evaluations in the literature. Final holdout results are reported separately as a stricter estimate of generalization. Stratification results correspond to the EX_PLUS_NON_EX_TOP setting.

### 4.6. SHAP-Based Explainability

To improve the interpretability of the proposed multimodal framework, SHAP analysis was performed on the CatBoost-based three-class survival stratification model. The analysis was designed to examine model behavior at three complementary levels: global feature importance, block-level modality contribution, and local patient-level explanations.

**Figure 4:**
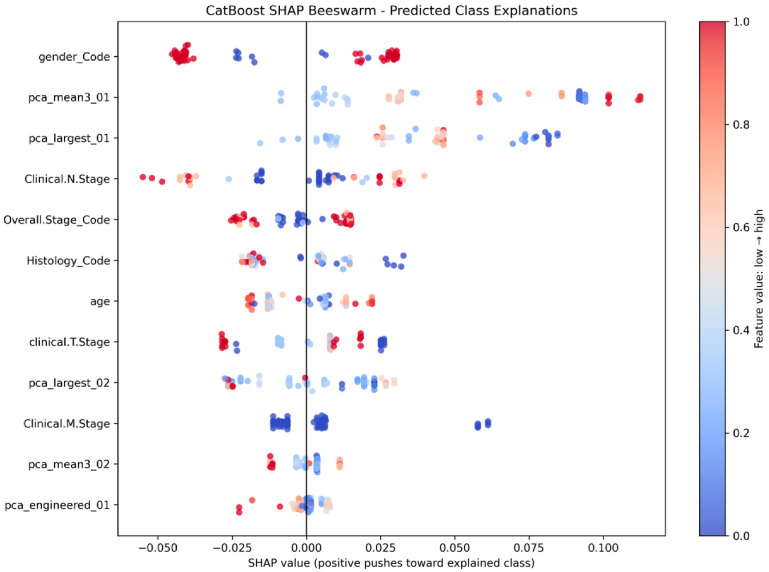
SHAP bee-swarm analysis. Abbreviations: pca_largest = largest-slice RadImageNet embedding PC; pca_mean3 = largest-slice ±1 mean embedding PC; pca_engineered = engineered imaging feature PC.

**Figure 5:**
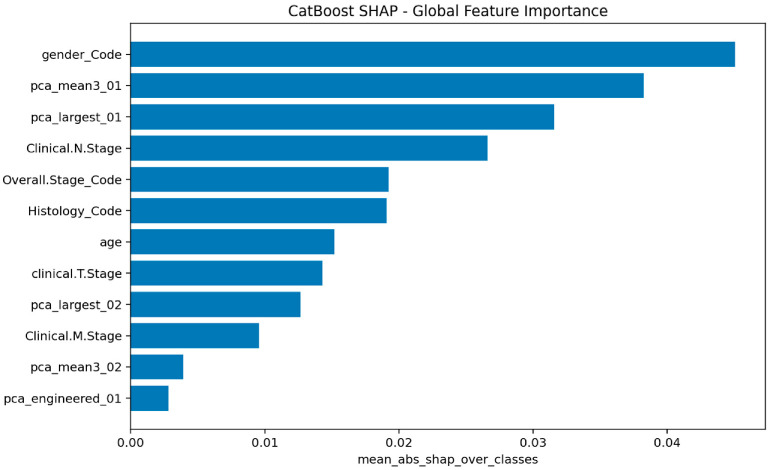
Global SHAP feature importance.

At the global level, the SHAP bee-swarm plot and the mean absolute SHAP ranking showed that the model relied primarily on clinical/tabular variables, while also making meaningful use of deep image embedding components derived from the CT-based ROI representations (see Figure 4 and Figure 5). Among the most influential variables were gender_Code, Clinical.N.Stage, Overall.Stage_Code, Histology_Code, age, and clinical.T.Stage, together with image-derived PCA components such as pca_mean3_01 and pca_largest_01. This indicates that the model did not depend on a single modality alone; instead, it combined dominant clinical signals with complementary information extracted from deep image embeddings.

**Figure 6:**
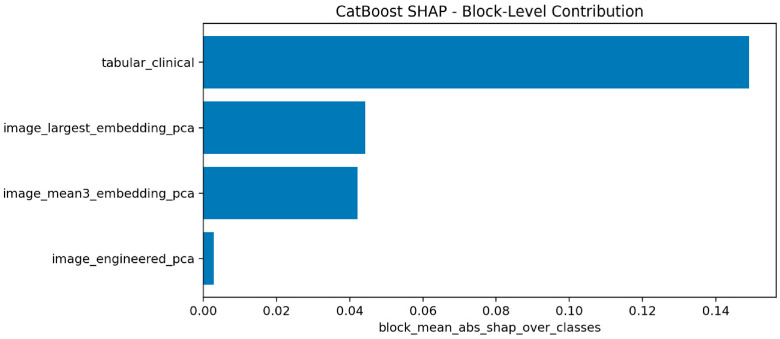
Block-level SHAP contribution analysis.

The block-level SHAP analysis further clarified the relative contribution of each modality group (Figure 6). The tabular clinical block showed the largest overall contribution by a clear margin, confirming that clinical information carried the strongest predictive signal in this dataset. However, both the largest-slice embedding PCA and top-3-slice mean embedding PCA blocks also contributed non-negligibly, whereas the engineered imaging feature block had only a limited contribution. This pattern supports the main interpretation of the study: clinical variables form the backbone of prediction, while compact deep image embeddings provide additional complementary information, and simple engineered ROI-based descriptors contribute less.

**Figure 7:**
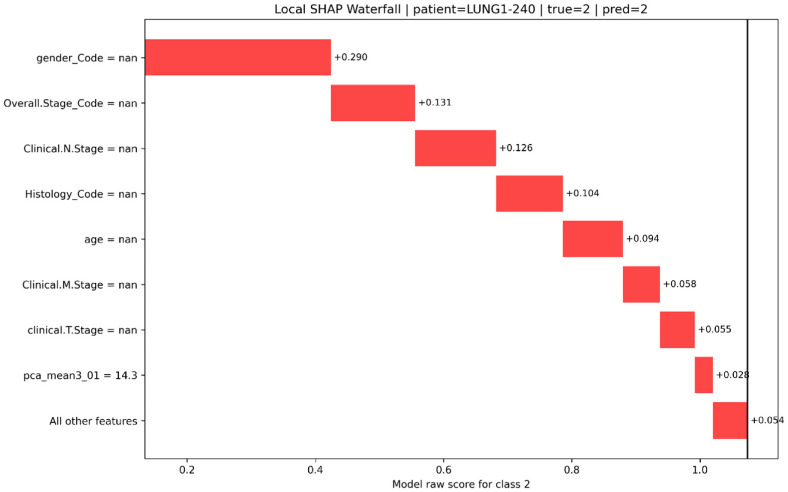
Local SHAP explanation for a correctly classified patient (LUNG1-240). The patient had an observed follow-up time of 3551 days and was correctly classified as long-survival under the EX_PLUS_NON_EX_TOP setting.

**Figure 8:**
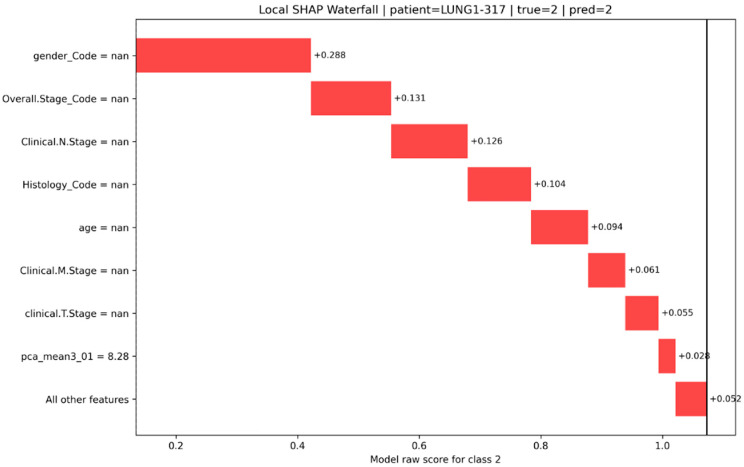
Local SHAP explanation for another correctly classified patient (LUNG1-317). The patient had a follow-up duration of 3362 days and was also correctly predicted as long-survival under the EX_PLUS_NON_EX_TOP setting.

**Figure 9:**
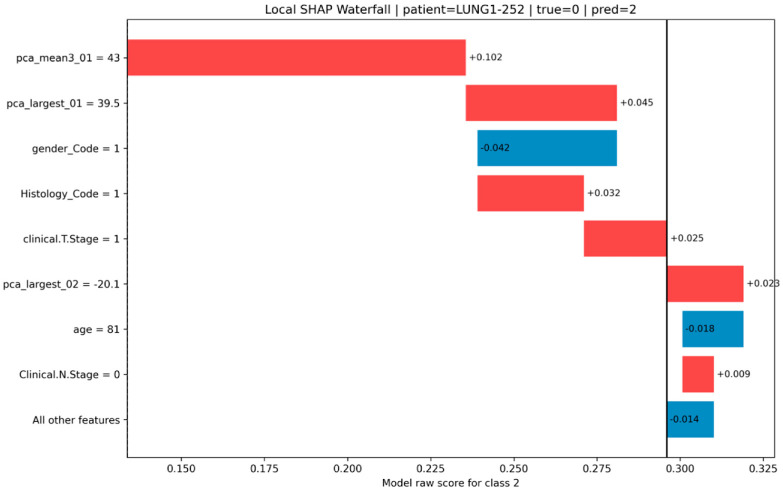
Local SHAP explanation for a misclassified patient (LUNG1-252). The patient had an observed survival time of 104 days but was predicted as a long-survival under the EX_PLUS_NON_EX_TOP setting.

To complement the global interpretation, local SHAP waterfall plots were examined for representative patients (Figure 7, Figure 8 and Figure 9). Two correctly classified examples from the long-survival class (Figure 7 and Figure 8) show that the predicted outcome is primarily supported by clinical variables, with additional positive contributions from image-derived embedding features. The consistency between these two cases indicates that the model relies on a stable combination of clinical and imaging-derived signals when identifying long-survival patients.

In contrast, the misclassified example (true short-survival, predicted long-survival; Figure 9) reveals that strong positive contributions from image embedding components and selected clinical variables collectively push the model toward the long-survival class, despite some offsetting negative contributions. This case highlights how competing signals across modalities can lead to incorrect predictions.

Overall, these local explanations demonstrate not only which features are important globally, but also how different modalities interact at the individual prediction level.

Taken together, the SHAP analysis supports three main conclusions. First, clinical/tabular variables were the dominant drivers of prediction. Second, deep image embedding features contributed a meaningful complementary signal, particularly the PCA-compressed representations derived from the largest tumor slice and the mean embedding of the top three slices. Third, engineered imaging features played a relatively minor role in the final model. These findings are consistent with the model interpretation results and support the use of a multimodal tabular framework in which structured clinical data provide the main prognostic signal, while compact learned image representations add complementary information.

## 5. Discussion

This study presents a data-efficient and explainable multimodal framework for NSCLC survival prediction under limited-sample conditions. Instead of training an end-to-end deep survival model directly on CT volumes, tumor-focused CT information was converted into compact tabular representations using a pretrained RadImageNet-InceptionV3 network and then combined with clinical variables. The final prediction models were trained using gradient-boosted trees, allowing the framework to benefit from deep image representations while preserving the advantages of structured tabular modeling.

This strategy was motivated by four main considerations:

(i) NSCLC survival prediction datasets are often limited in size, heterogeneous, and affected by censoring, making fully data-driven end-to-end deep learning approaches vulnerable to overfitting and unstable generalization [11,29];
(ii) gradient-boosted tree models remain highly competitive for structured tabular data, especially in limited-sample settings where they often outperform deep neural networks on tabular prediction tasks [19,20];
(iii) although survival time is naturally a continuous outcome, many NSCLC survival prediction studies formulate the problem through risk scores, survival-group stratification, or time-dependent classification rather than direct prediction of survival time in days;
(iv) while multimodal survival prediction studies combining imaging and clinical data exist, fewer studies convert pretrained deep CT representations into a compact tabular feature space and evaluate them together with clinical variables using boosted-tree models under a strict final holdout validation protocol.

The primary analysis modeled survival time directly as a continuous regression target. This is a stricter and more demanding formulation than survival-group classification, because the model is required to preserve information on survival duration rather than only assign patients to broad prognostic categories. On the strictly held-out final validation set, the best continuous regression model achieved a C-index of 0.576. This result should be interpreted in the context of the task definition and validation design: the final validation cohort was not used during feature processing, hyperparameter tuning, model selection, or cross-validation.

To enable comparison with the survival-group and risk-stratification formulations commonly used in the literature, an auxiliary three-class survival stratification analysis was also performed. In the EX_PLUS_NON_EX_TOP setting, where non-event patients were assigned to the upper survival class, the development-stage score-based class C-index reached 0.690 with CatBoost, 0.688 with XGBoost, and 0.673 with LightGBM. These values are competitive with several recent radiomics, deep-radiomics, and multimodal survival prediction studies, despite the smaller cohort size and compact feature representation used in the present work. On the separate final validation set, the best score-based class C-index remained 0.655, providing a more conservative yet more realistic estimate of generalization.

The distinction between continuous regression and direct multiclass classification is important. Direct classification naturally produced stronger class-label metrics, such as accuracy and macro-F1, because the models were optimized directly for survival-group assignment. Regression-derived stratification, however, preserves the link to real-valued survival prediction and therefore provides a different view of model behavior. These two analyses should not be treated as interchangeable. The classification-style results allow comparison with prior survival stratification studies, while the regression results support the main methodological position that survival prediction was not reduced only to coarse risk grouping.

The results also indicate that clinical variables carried the dominant prognostic signal in this cohort. This is expected in NSCLC survival prediction, where age, stage-related descriptors, histology, and other patient-level variables are clinically meaningful predictors. The role of image-derived features in the proposed framework was not to replace these clinical predictors, but to add tumor-focused imaging information in a compact and model-compatible form. Block-wise PCA reduced the high-dimensional RadImageNet embeddings to a small number of components, allowing them to be integrated with clinical variables without overwhelming the limited sample size.

The SHAP analysis further supports this interpretation. Global SHAP results showed that clinical variables were the strongest contributors to the final CatBoost stratification model. At the same time, PCA-compressed deep image embedding components derived from the largest tumor slice and the best-slice ±1 mean representation also contributed to the predictions. Block-level SHAP analysis confirmed that the largest contribution came from tabular clinical variables, followed by learned image embedding components, while engineered imaging features contributed less. This suggests that pretrained deep image embeddings captured complementary information beyond simple ROI-based geometric or statistical descriptors. A focused feature-group contribution analysis using CatBoost on the final validation set yielded a score-based class C-index of 0.631 for the clinical-only model, 0.553 for the imaging-only model, and 0.655 for the full multimodal model (Supplementary Table S2). These results confirm that clinical variables provided the dominant prognostic signal, while CT-derived imaging features contributed a modest but measurable improvement when combined with clinical information. The relatively high SHAP contribution of gender_Code should be interpreted cautiously, because SHAP reflects model-learned associations within this cohort rather than causal prognostic effects. Given the higher proportion of male patients in the final validation set, this finding may partly reflect a cohort-specific association or distributional imbalance and should be reassessed in larger external cohorts before drawing clinical conclusions about sex-related survival differences.

Local SHAP explanations provided an additional patient-level view of model behavior. In correctly classified long-survival examples, the model prediction was mainly supported by clinical variables, with additional contributions from image-derived embedding components. In the misclassified example, competing contributions from clinical and imaging-derived features pushed the model toward an incorrect long-survival prediction. These local explanations are useful because they make the decision process inspectable and show how different feature groups interact in individual cases.

Nevertheless, compared with recent multimodal NSCLC survival prediction studies, the present work should be interpreted as a proof-of-concept demonstration of a compact image-to-tabular survival modeling pipeline rather than as a fully validated clinical survival prediction system. Under the literature-comparable EX_PLUS_NON_EX_TOP stratification setting, the development-stage score-based class C-index reached 0.690 with CatBoost, while the final validation score-based class C-index was 0.655. These values remain meaningful in view of the limited cohort size, compact 12-feature tabular representation, and separation of the final validation set from model development. The continuous regression result should be interpreted separately: the best final validation event-patient C-index of 0.576 reflects the more demanding task of predicting survival time directly in days, and therefore should not be compared directly with classification-style survival stratification metrics commonly reported in the literature.

The findings should also be interpreted in light of the study scope. The analysis was based on a single public dataset, without independent external validation, and relied on available tumor segmentation masks. The representative largest-slice ±1 strategy captured only a subset of the full segmented tumor volume; this was a deliberate data-efficient design choice, but future work should compare it with full 3D tumor embeddings, voxel-level spatial graph representations, or hybrid CNN-GNN models when larger datasets are available. The 303- and 703-day thresholds used for auxiliary survival stratification were development-cohort tertile-based cut-points; although appropriate for creating balanced short-, intermediate-, and long-survival groups for internal evaluation, they may not directly correspond to clinically standardized NSCLC risk cut-offs and should be reassessed in external cohorts or replaced by clinically defined time horizons when appropriate. Scanner, vendor, acquisition protocol, and reconstruction variability were not explicitly modeled, and the available clinical variables did not include potentially relevant prognostic factors such as ECOG performance status, treatment details, actionable genomic alterations, comorbidities, or laboratory biomarkers. In addition, categorical clinical variables were represented using compact integer-based encoding rather than one-hot encoding; this was a deliberate small-data design choice to avoid feature expansion and maintain a consistent preprocessing pipeline across CatBoost, XGBoost, and LightGBM, but alternative encoding strategies should be evaluated in future studies. These limitations do not diminish the methodological value of the proposed framework, but they indicate that external multicenter validation with richer clinical, imaging, and treatment-related metadata is required before clinical translation can be considered. Because the final validation cohort was limited in size, the reported C-index values should be interpreted with statistical uncertainty and should not be regarded as definitive population-level performance estimates. Larger independent external validation cohorts are required for more stable estimation of generalizability.

Although graph-based medical AI methods were outside the scope of the present study, they may also represent a possible future extension for richer relational modeling. Recent studies have explored graph neural networks for medical and relational learning tasks [30,31]. In principle, future NSCLC studies could investigate whether tumor-region graphs, voxel-level spatial graphs, or clinical knowledge graphs provide additional prognostic information beyond the compact image-to-tabular representation used here. However, the primary objective of the present work was deliberately more pragmatic: developing a compact, data-efficient, and explainable multimodal framework suitable for limited-sample settings.

Taken together, the findings support the central methodological claim of the study: pretrained deep CT representations can be transformed into compact tabular features and modeled together with clinical variables using gradient-boosted trees. Within the limitations of a single-dataset proof-of-concept study, this provides a feasible, interpretable, and data-efficient framework for NSCLC survival prediction under limited-sample conditions.

## 6. Conclusions

This study presents a data-efficient and explainable multimodal framework for NSCLC survival prediction using CT-derived deep embeddings, engineered imaging features, and clinical variables. Instead of training an end-to-end deep survival model on CT volumes, tumor-focused image information was converted into compact tabular representations using a pretrained RadImageNet-InceptionV3 model and combined with structured clinical data. This allowed gradient-boosted tree models to be applied in a small-sample setting while preserving interpretability and computational efficiency.

The results show that the proposed framework can achieve competitive survival stratification performance under literature-comparable evaluation conditions, while also reporting a stricter final holdout validation estimate. In the auxiliary three-class survival stratification setting, the model achieved development-stage score-based class C-index values up to 0.690, and a final holdout score-based class C-index of 0.655. In parallel, the primary continuous regression analysis evaluated survival time directly in days and provided a more demanding assessment of real-valued survival prediction. This dual evaluation is important because it distinguishes survival-group stratification from direct survival-time regression, two tasks that are often treated too similarly in the literature.

The SHAP analysis confirmed that clinical variables provided the strongest prognostic signal in this cohort. At the same time, PCA-compressed deep image embedding components contributed complementary information, especially those derived from the largest tumor slice and the multi-slice mean representation. These findings support the use of a compact image-to-tabular fusion strategy in which clinical data form the main predictive backbone, while learned CT-derived representations add tumor-focused information in an interpretable form.

Future work should evaluate the proposed framework on independent external NSCLC cohorts to assess generalization across institutions, scanners, vendors, acquisition protocols, and treatment settings. Further studies should also incorporate richer clinical variables, including performance status, treatment information, genomic markers, comorbidities, and laboratory biomarkers where available. In addition, the reliance on predefined tumor masks should be addressed by evaluating segmentation variability or integrating automated segmentation pipelines. From the imaging perspective, richer representations may also be explored, including more complete 3D tumor embeddings, voxel-level graph representations, and hybrid CNN-GNN architectures that combine pretrained image embeddings with spatial message passing across tumor regions. Clinical knowledge graphs may further support the incorporation of structured prior knowledge and provide explanations that go beyond feature-level attribution. These extensions could strengthen the clinical and relational context of the proposed framework while preserving its data-efficient, explainable, and tabular-learning-oriented design.

Overall, this study supports a practical alternative to fully end-to-end deep survival modeling for small medical imaging cohorts. By combining pretrained CT embeddings, clinical variables, gradient-boosted trees, strict validation, and SHAP-based explainability, the proposed framework offers a robust and interpretable direction for NSCLC survival prediction under limited-data conditions.

## Figures and Tables

**Table 1:** Baseline characteristics of the development and final validation sets.

Characteristic	Development Set	Final Validation Set
All patients, n	302	75
Age, mean ± SD	67.4 ± 9.6	69.7 ± 10.0
Age, median [IQR]	67.9 [61.1–74.9]	68.3 [63.7–76.9]
Male sex, n (%)	202 (66.9%)	64 (85.3%)
Event observed, n (%)	268 (88.7%)	67 (89.3%)
Non-event/censored, n (%)	34 (11.3%)	8 (10.7%)
Event patients with clinical variables, n	268	67
Clinical T stage 1–2, n (%)	160 (59.7%)	44 (65.7%)
Clinical T stage 3–4/other, n (%)	108 (40.3%)	23 (34.3%)
Clinical N stage 0, n (%)	105 (39.2%)	32 (47.8%)
Clinical N stage ≥ 1, n (%)	163 (60.8%)	35 (52.2%)
Clinical M stage 0, n (%)	264 (98.5%)	66 (98.5%)
Overall stage I–II, n (%)	85 (31.7%)	24 (35.8%)
Overall stage III–IV, n (%)	183 (68.3%)	43 (64.2%)
Histology code 2–3, n (%)	178 (66.4%)	36 (53.7%)
Other histology codes, n (%)	90 (33.6%)	31 (46.3%)

**Table 2:** Continuous survival regression performance on development cross-validation and final holdout validation sets.

Model	Dev CV C-Index	Dev CV RMSE	Dev CV MAE	Dev CV RMSE-Log	Dev CV MAE-Log	Dev CV Pearson	Holdout C-Index	Holdout RMSE	Holdout MAE	Holdout RMSE-Log	Holdout MAE-Log	Holdout Pearson
LightGBM	0.605	738.3	468.9	0.974	0.778	0.221	0.576	866.7	545.3	1.116	0.886	0.201
CatBoost	0.596	745.4	475.9	0.986	0.793	0.206	0.564	875.4	547.0	1.121	0.890	0.182
XGBoost	0.600	747.7	477.1	0.988	0.796	0.212	0.556	879.8	551.8	1.129	0.900	0.212

**Table 3:** Class distribution for the auxiliary three-class survival stratification task.

Setting	Split	Short Survival	Intermediate Survival	Long Survival	Total
EX_ONLY	Development	88	91	89	268
EX_ONLY	Final validation	23	19	25	67
EX_PLUS_NON_EX_TOP	Development	88	91	123	302
EX_PLUS_NON_EX_TOP	Final validation	23	19	33	75

Note: In the EX_PLUS_NON_EX_TOP setting, non-event/censored patients were assigned to the long-survival class by design.

**Table 4:** Three-class survival stratification performance (development CV and final holdout validation).

**(A) EX_PLUS_NON_EX_TOP**							
**Model**	**Dev Accuracy**	**Dev F1-Macro**	**Dev Class C-Index**	**Holdout Accuracy**	**Holdout F1-Macro**	**Holdout Class C-Index**	**Holdout Days C-Index**
CatBoost	0.487	0.404	0.690	0.440	0.374	0.655	0.559
XGBoost	0.454	0.406	0.688	0.440	0.387	0.645	0.548
LightGBM	0.464	0.414	0.673	0.467	0.429	0.639	0.548
**(B) EX_ONLY**							
**Model**	**Dev Accuracy**	**Dev F1-Macro**	**Dev Class C-Index**	**Holdout Accuracy**	**Holdout F1-Macro**	**Holdout Class C-Index**	**Holdout Days C-Index**
CatBoost	0.414	0.376	0.637	0.388	0.362	0.531	0.548
XGBoost	0.396	0.374	0.636	0.418	0.397	0.558	0.548
LightGBM	0.369	0.365	0.628	0.433	0.433	0.525	0.543

**Table 5:** Regression-derived versus direct three-class survival stratification on the final holdout validation set.

**(A) EX_PLUS_NON_EX_TOP**							
**Model**	**Approach**	**Accuracy**	**F1-Macro**	**Class C-Index (Score)**	**Class C-Index (Hard)**	**Event-days C-Index (Score)**	**Event-Days C-Index (Hard)**
CatBoost	Regression-derived	0.253	0.135	0.633	0.500	0.564	0.500
CatBoost	Direct classification	0.440	0.374	0.655	0.557	0.559	0.526
XGBoost	Regression-derived	0.253	0.135	0.570	0.500	0.556	0.500
XGBoost	Direct classification	0.440	0.387	0.645	0.571	0.548	0.526
LightGBM	Regression-derived	0.307	0.225	0.654	0.536	0.576	0.514
LightGBM	Direct classification	0.467	0.429	0.639	0.590	0.548	0.538
**(B) EX_ONLY**							
**Model**	**Approach**	**Accuracy**	**F1-Macro**	**Class C-Index (Score)**	**Class C-Index (Hard)**	**Event-Days C-Index (Score)**	**Event-Days C-Index (Hard)**
CatBoost	Regression-derived	0.284	0.147	0.582	0.500	0.564	0.500
CatBoost	Direct classification	0.388	0.362	0.566	0.531	0.548	0.532
XGBoost	Regression-derived	0.284	0.147	0.567	0.500	0.556	0.500
XGBoost	Direct classification	0.418	0.397	0.557	0.558	0.548	0.543
LightGBM	Regression-derived	0.328	0.231	0.597	0.528	0.576	0.514
LightGBM	Direct classification	0.433	0.433	0.551	0.525	0.543	0.529

**Table 6:** Comparison with selected survival prediction studies and the present framework.

Study	N	Modalities	Task	Protocol	C-Index
Le et al. (2025) [18]	420/516	CT + radiomics + clinical	Survival (DeepSurv)	Train/test (Lung1 → Lung2)	0.733/0.751
Hou et al. (2022) [28]	492	CECT + radiomics + clinical	Survival (DeepSurv)	Test/time-dependent	0.74–0.75
Yolchuyeva et al. (2023) [11]	385	CT radiomics + clinical	Survival/classification	External validation	0.57 (val)
SurvPGC (Hou et al., 2025) [29]	354/1035/298	Pathology + genomics + clinical	Prognosis	TCGA validation	0.67–0.70
This study (dev, comparable)	302	CT embeddings + imaging + clinical	3-class stratification	Development-stage	0.690 (CatBoost)
This study (final holdout)	75	CT embeddings + imaging + clinical	3-class stratification	Strict holdout	0.655 (CatBoost)
This study (regression)	67	CT embeddings + imaging + clinical	Survival regression	Strict holdout	0.576 (LightGBM)

## Data Availability

The NSCLC-Radiomics/LUNG1 dataset used in this study is publicly available through The Cancer Imaging Archive (TCIA). The derived feature matrices, split assignments, selected model parameters, and analysis scripts are available from the corresponding author upon reasonable request for academic reproducibility purposes.

## Supplementary Materials

The following supporting information can be downloaded at: https://www.mdpi.com/article/10.3390/diagnostics16121941/s1, Figure S1: Diagnostic visualization of model calibration and observed-versus-predicted survival; Table S1: Hyperparameter tuning details and selected model settings; Table S2: Feature-group contribution analysis using CatBoost on the final validation set.

## Abbreviations

The following abbreviations are used in this manuscript:

NSCLC	Non-small cell lung cancer
CT	Computed tomography
CNNs	Convolutional neural networks
SHAP	SHapley Additive exPlanations
TNM	Tumor-node-metastasis
HU	Hounsfield units
PCA	Principal component analysis
ROI	Region of interest
RMSE	Root mean squared error
MAE	Mean absolute error
RMSE-log	Root mean squared logarithmic error
MAE-log	Mean absolute logarithmic error
