# Supplementary material for: Data-Efficient and Explainable Multimodal Survival Prediction in NSCLC Using Deep Image Embeddings, Clinical Variables, and Gradient-Boosted Trees
Source: Diagnostics (Basel). 2026 Jun 22;16(12):1941. doi: 10.3390/diagnostics16121941 (PMC13298473; doi:10.3390/diagnostics16121941)
Supplement: Supplementary file 1 [file diagnostics-16-01941-s001.zip › diagnostics-4334864-supplementary.pdf]

diagnostics

MDPI

Supplementary Table S1. Hyperparameter tuning details and selected model settings.

Model	Tuned parameters	Selected values	Early stopping
CatBoost	iterations, learning_rate, depth, l2_leaf_reg, subsample	iterations=38, learning_rate=0.05, depth=2, l2_leaf_reg=10.0, subsample=0.50	No
XGBoost	n_estimators, learning_rate, max_depth, reg_lambda, subsample	n_estimators=55, learning_rate=0.01, max_depth=2, reg_lambda=1.0, subsample=0.55	No
LightGBM	n_estimators, learning_rate, max_depth, reg_lambda, subsample	n_estimators=50, learning_rate=0.03, max_depth=4, reg_lambda=3.0, subsample=0.50	No

Main software environment: TensorFlow 2.21.0, NumPy 2.3.5, Pandas 2.2.3, pydicom 3.0.1. CatBoost 1.2.8, XGBoost 2.1.3, LightGBM 4.6.0, and scikit-learn 1.6.1 were used for gradient-boosted modeling, preprocessing, PCA, and cross-validation.

Supplementary Figure S1. Diagnostic visualization of model calibration and observed-versus-predicted survival.

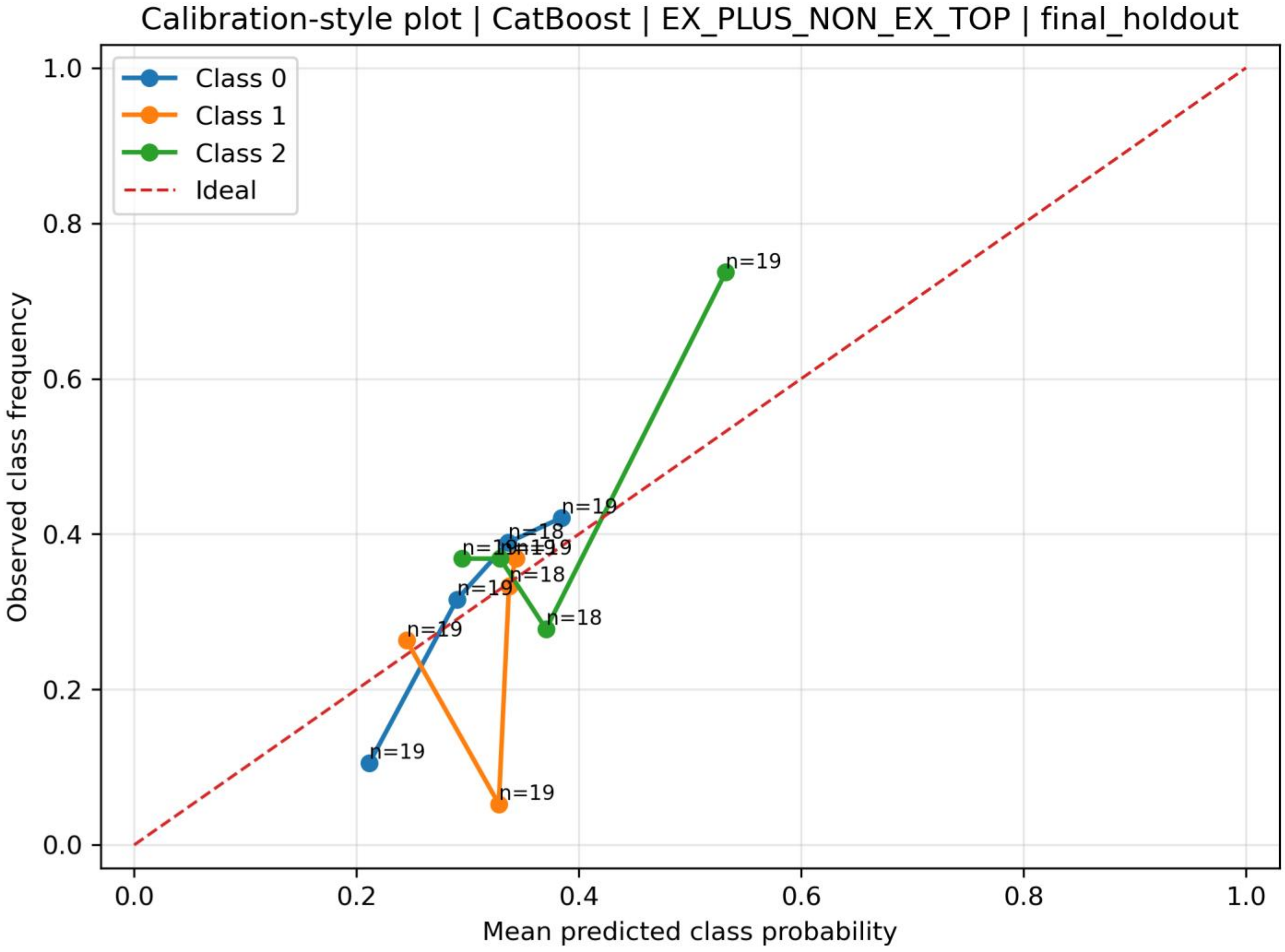

(A) Calibration-style plot for the CatBoost EX_PLUS_NON_EX_TOP three-class stratification model on the final holdout set.

https://doi.org/10.3390/diagnostics16121941

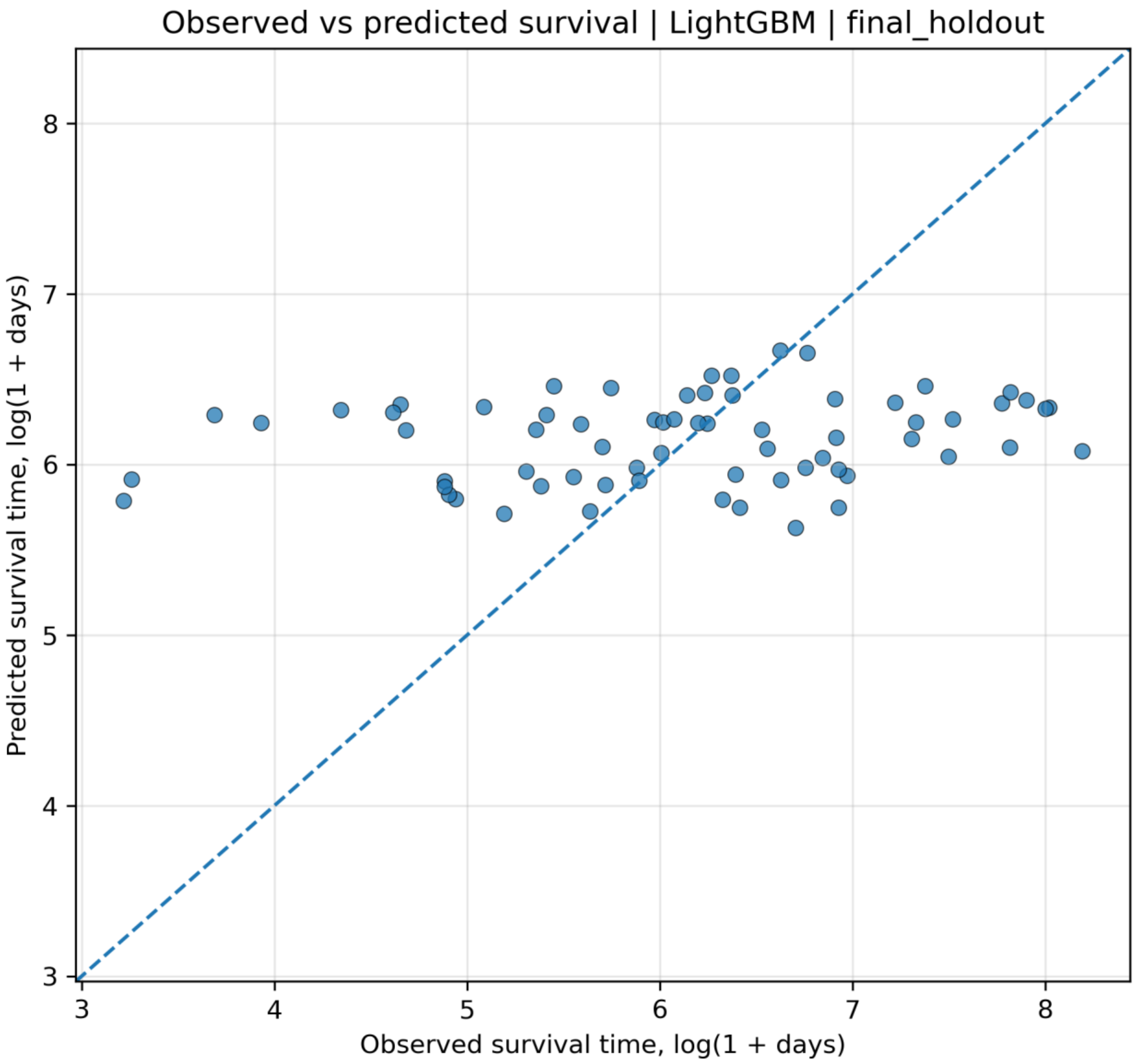

(B) Observed-versus-predicted survival time for the LightGBM continuous regression model on final holdout event patients, shown on a log(1 + days) scale.

Supplementary Table S2. Feature-group contribution analysis using CatBoost on the final validation set.

Feature group	Input features	Holdout score-based class C-index
Clinical-only	Clinical variables only	0.631
Imaging-only	Engineered imaging features + CT embedding PCA components	0.553
Full multimodal	Clinical variables + engineered imaging features + CT embedding PCA components	0.655

https://doi.org/10.3390/diagnostics16121941
